# Inhibition of AdeB, AceI, and AmvA Efflux Pumps Restores Chlorhexidine and Benzalkonium Susceptibility in *Acinetobacter baumannii* ATCC 19606

**DOI:** 10.3389/fmicb.2021.790263

**Published:** 2022-02-07

**Authors:** Antonella Migliaccio, Eliana Pia Esposito, Maria Bagattini, Rita Berisio, Maria Triassi, Eliana De Gregorio, Raffaele Zarrilli

**Affiliations:** ^1^Department of Public Health, University of Naples Federico II, Naples, Italy; ^2^Institute of Biostructures and Bioimaging, National Research Council, Naples, Italy; ^3^Department of Molecular Medicine and Medical Biotechnology, University of Naples Federico II, Naples, Italy

**Keywords:** *Acinetobacter baumannii*, chlorhexidine susceptibility, efflux pumps, AdeB, biofilm growth, resveratrol, piperine, benzalkonium

## Abstract

The management of infections caused by *Acinetobacter baumannii* is hindered by its intrinsic tolerance to a wide variety of biocides. The aim of the study was to analyze the role of different *A. baumannii* efflux pumps (EPs) in tolerance to chlorhexidine (CHX) and benzalkonium (BZK) and identify non-toxic compounds, which can restore susceptibility to CHX and BZK in *A. baumannii*. *A. baumannii* ATCC 19606 strain was tolerant to both CHX and BZK with MIC and MBC value of 32 mg/L. CHX subMIC concentrations increased the expression of *adeB* and *adeJ* (RND superfamily), *aceI* (PACE family) and *amvA* (MFS superfamily) EP genes. The values of CHX MIC and MBC decreased by eightfold in Δ*adeB* and twofold in Δ*amvA* or Δ*aceI* mutants, respectively, while not affected in Δ*adeJ* mutant; EPs double and triple deletion mutants showed an additive effect on CHX MIC. CHX susceptibility was restored in double and triple deletion mutants with inactivation of *adeB* gene. BZK MIC was decreased by fourfold in Δ*adeB* mutant, and twofold in Δ*amvA* and Δ*aceI* mutants, respectively; EPs double and triple deletion mutants showed an additive effect on BZK MIC. BZK susceptibility was recovered in Δ*adeB* Δ*aceI* Δ*adeJ* and Δ*amvA* Δ*adeB* Δ*adeJ* triple mutants. The structural comparison of AdeB and AdeJ protomers showed a more negatively charged entrance binding site and F-loop in AdeB, which may favor the transport of CHX. The carbonyl cyanide m-chlorophenylhydrazine protonophore (CCCP) EP inhibitor reduced dose-dependently CHX MIC in *A. baumannii* ATCC 19606 and in Δ*adeJ*, Δ*aceI*, or Δ*amvA* mutants, but not in Δ*adeB* mutant. Either piperine (PIP) or resveratrol (RV) at non-toxic concentrations inhibited CHX MIC in *A. baumannii* ATCC 19606 parental strain and EPs gene deletion mutants, and CHX-induced EP gene expression. Also, RV inhibited BZK MIC and EP genes expression in *A. baumannii* ATCC 19606 parental strain and EPs mutants. These results demonstrate that tolerance to CHX and BZK in *A. baumannii* is mediated by the activation of AdeB, AceI and AmvA EPs, AdeB playing a major role. Importantly, inhibition of EP genes expression by RV restores CHX and BZK susceptibility in *A. baumannii.*

## Introduction

Bacteria belonging to the genus *Acinetobacter* are glucose non-fermentative Gram-negative coccobacilli that are a frequent cause of health-care associated infections and hospital outbreaks. *A. baumannii* represents the most clinically relevant species among those belonging to the *A. baumannii*-*calcoaceticus* group (Wong et al., 2017). Global epidemiology of *A. baumannii* shows a clonal population structure dominated by two major international clonal lineages and few additional epidemic clones (Gaiarsa et al., 2019). The most successful *Acinetobacter* clones show resistance to a broad range of antimicrobials and tolerance to disinfectants and share virulence features such as biofilm formation on biotic and abiotic surfaces, resistance to desiccation and adherence to epithelial cells (Giannouli et al., 2013; Wong et al., 2017; Harding et al., 2018). *A. baumannii* strains responsible for nosocomial outbreaks are resistant to a wide range of antimicrobials, resistance to carbapenems being present in more than 90% of them and resistance to colistin emerging also (Wong et al., 2017).

*A. baumannii* persistence in the contaminated hospital environment is contributed also by reduced susceptibility of the bacteria to a broad range of biocides used as antiseptics or disinfectants, such as the bisphenol triclosan (TRI), the quaternary ammonium compounds benzalkonium chloride (BZK), dequalinium chloride (DQ), and cetrimide (CT), and the biguanide chlorhexidine (CHX) (McDonnell and Russell, 1999). CHX is a positively charged molecule able to react with the negatively charged microbial cell surface, thereby destroying the integrity of the cell membrane (McDonnell and Russell, 1999). CHX is a bactericidal agent, which is widely used for hand hygiene, skin antisepsis, oral care, and patient washing (Milstone et al., 2008). BZK has been widespread used as disinfectant in hospitals, food industry and commercial products, or antiseptic in antimicrobial soaps (Merchel Piovesan Pereira and Tagkopoulos, 2019). Reduced susceptibility to CHX and BZK is emerging in various nosocomial pathogens (Kampf, 2016; Merchel Piovesan Pereira and Tagkopoulos, 2019; Weber et al., 2019). Reduced susceptibility to CHX in *A. baumannii* has been correlated with activation of different efflux systems (Rajamohan et al., 2010a,b; Hassan et al., 2013; Tucker et al., 2014; Du et al., 2018; Harding et al., 2018; Kornelsen and Kumar, 2021). In particular, activation of AdeB and AdeJ resistance–nodulation–cell division (RND) efflux systems (Rajamohan et al., 2010a; Tucker et al., 2014), AmvA and CraA major facilitator superfamily (MFS) efflux systems (Rajamohan et al., 2010b; Foong et al., 2019) have been shown to induce tolerance to CHX and other disinfectants in clinical *A. baumannii* isolates. Reduced susceptibility to chlorhexidine has also been associated with activation of AceI proteobacterial antimicrobial compound efflux (PACE) system in *A. baumannii* ATCC17978 (Hassan et al., 2013; Tucker et al., 2014).

Non-toxic natural substances such as the alkaloid piperine (Haq et al., 2021) and the monomeric stilbenoid resveratrol (Mattio et al., 2020) are able to modulate the susceptibility to CHX in *A. baumannii* and other bacteria (Sharma et al., 2010; Mirza et al., 2011; Singkham-In et al., 2020).

The objectives of the present study were to: (i) study the contribution of efflux pump systems to and the molecular mechanisms responsible for tolerance to CHX and BZK in *A. baumannii*; (ii) identify non-toxic compounds, which can modulate and restore susceptibility to CHX and BZK in *A. baumannii*.

## Materials and Methods

### Bacterial Strain, Growth Condition, Antibiotics, and Reagents

*A. baumannii* ACICU (Iacono et al., 2008), *A. baumannii* AYE (Poirel et al., 2003), *A. baumannii* ATCC 19606 (Janssen et al., 1997), *Escherichia coli* 25922 and *E. coli* S17 λpir (Simon et al., 1983) strains were used for this study. *E. coli* ATCC 25922 was purchased from LGC Standards S.r.l., Italy). All strains were cultured under aerobic conditions at 37°C in Luria-Bertani (LB) broth/agar. LB broth, cation-adjusted Mueller-Hinton broth (CAMHB) and Tryptic soy broth (TSB) were used to perform growth curves, susceptibility tests and biofilm assays. The chemical reagents were chlorhexidine digluconate (CHX), carbonyl cyanide m-chlorophenylhydrazine (CCCP), triclosan (5-chloro-2-(2,4-dichlorophenoxy) phenol (TRI), the quaternary ammonium compounds benzalkonium chloride (alkylbenzyldimethylammonium chloride (BZK), dequalinium chloride (DQ), and cetrimide (alkyltrimethylammonium bromide (CT), piperine (1-piperoyliperidine, PIP) and resveratrol (3,5,4’-trihydroxy-*trans*-stilbene, RV). The antimicrobials and chemical reagents were purchased from Sigma-Aldrich (Sigma, Milan, Italy).

### Construction of *adeB*, *adeJ*, *aceI*, and *amvA* Gene Knockouts

DNA and plasmid DNAs of *A. baumannii* ATCC 19606 and knockout mutants were extracted using the DNeasy Blood & Tissue Kit (Qiagen, Milan, Italy) and the Plasmid Mini/Midi Kits (Qiagen, Milan, Italy), respectively, according to the manufacturer’s instructions. *A. baumannii* ATCC 19606 was mutagenized as previously described (Amin et al., 2013; De Gregorio et al., 2015) with the following minor changes. The upstream and downstream fragments of target genes were amplified using the primers listed in Supplementary Table 1 and inserted in the TA Cloning pCR2.1 vector (Invitrogen); 100 μL of competent *E. coli* DH5α were transformed with TA-cloning vector. The upstream fragments were digested with *Not*I*-Bam*HI and cloned into suicide vector pMo130-Tel^R^, creating pMo130-TelR-Up. Next, the downstream fragments were digested with *Bam*HI*–Sph*I and inserted in pMo130-TelR-Up to obtain the plasmid pMo130-TelR-Up/Dw. The final plasmid was introduced into *E. coli* S17-1 λpir by CaCl_2_ transformation and mobilized to the *A. baumannii* ATCC 19606 strain or single/double mutants via conjugation as described (Amin et al., 2013), to obtain single, double and triple mutants. Transconjugants were selected in LB agar containing 30 mg/L tellurite + 50 mg/L ampicillin and 50 mg/L kanamycin + 50 mg/L ampicillin, cultured in LB broth containing 14% sucrose. Serial dilutions were spread onto LB plates containing 14% sucrose. Colonies were screened for tellurite sensitivity to monitor excision of the suicide vector. The inactivation of *adeB*, *adeJ*, *aceI* and *amvA* genes were confirmed by PCR amplification using control primers (Supplementary Table 1).

### Determination of Minimum Inhibitory Concentration and Minimum Bactericidal Concentration

*A. baumannii* ATCC 19606 was grown overnight at 37°C on LB broth, under shaking (200 rpm). The MIC and MBC of CHX was determined by a manual microdilution method according to the recommended procedures by the European Committee for Antimicrobial Susceptibility Testing (Eucast) of the European Society of Clinical Microbiology and Infectious Diseases (Escmid) (2000) and the Clinical and Laboratory Standards (CLSI, 2019). Susceptibility was assessed to MIC value < 4 mg/L as described (Rajamohan et al., 2010a). *A. baumannii* ATCC 19606 and deletion mutants were grown on CAMHB at 37°C for 24 h. Afterward, 50 μL of 1 × 10^6^ CFU/mL bacterial cells were added to each well of the microtiter plate containing 50 μL of the CAMHB with twice the final concentration of molecules studied. Then the plates were incubated at 37°C for 18–24 h. Non-treated bacteria were used as controls. All tests were performed in triplicate and repeated three times.

### *In vitro* Combination Studies

The tests were carried out using the checkerboard method according to the previously reported method (Hall et al., 1983). Serial dilutions of CHX (0.5–164 mg/L) were prepared and combined with serial dilutions of piperine (8–128 mg/L), resveratrol (32–128 mg/L), CCCP (0.5, 1, and 2 mg/L). Subsequently, 1 × 10^6^ CFU/mL of either *A. baumannii* ATCC 19606 or deletion mutants were added to each well of the microtiter plate. Then the plates were incubated at 37°C for 18–24 h. All experiments were repeated three times.

### Biofilm Assay

Biofilm formation was examined using a crystal violet (CV) staining assay according to the previously reported method (De Gregorio et al., 2020). Bacterial cell suspension was prepared at 0.5 McFarland standard and it was diluted 1:100 in TSB. Subsequently, 100 μL of 1 × 10^6^ cells/mL was transferred into a 96-well flat-bottomed polystyrene microtiter plate containing 100 μL of scalar doses of CHX (164–0.5 g mg/L) and incubated at 37°C for 24 h. Non-treated bacteria were incubated with 100 μL of broth and used as the control. The culture supernatant was gently discarded, the wells were washed twice with phosphate-buffered saline (PBS) 1 × pH 7.4 and the biofilms were stained with 200 μL of 0.1% crystal violet for 20 min. The wells were washed twice with PBS 1X, and dye was re-eluted with 100% ethanol. The absorbance was measured at 595 nm using a microplate reader (Bio-Rad Laboratories S.r.l.). The OD595/OD600 ratio was used to normalize the amount of biofilm formed to the total cell content.

### RNA Purification and Real-Time RT-PCR

*A. baumannii* ATCC 19606 cells were grown over night on LB broth at 37°C at 200 rpm. Subsequently, ATCC 19606 was diluted 1:100 in LB broth alone or LB broth with subMIC of CHX or RV or PIP or CHX plus RV or CHX plus PIP and grown at 37°C at 200 rpm for a further 3 h to reach the exponential phase (OD_600_ = 0.5). Total RNA was isolated from three independent cultures according to the previously reported method (De Gregorio et al., 2018). The cDNAs were synthesized using QuantiTect Reverse Transcription Kit (Qiagen, Milan, Italy), according to the manufacturer’s protocol. Real-time RT-PCR assays were performed using SYBR Green master mix (Applied Biosystems) (Martinucci et al., 2016). The *rpoB* gene (the housekeeping gene) was used to normalize the expressions of target genes. The fold-change of the gene expression level was calculated using the 2^–^*^ΔΔ^*^ct^ method (Livak and Schmittgen, 2001). All experiments were performed three times in triplicate. The primers used in the qRT-PCR experiments were reported in Supplementary Table 2.

### Statistical Analysis

All statistical analyses were carried out using GraphPad Prism version 8.0 for Windows (GraphPad Software, San Diego, CA, United States). All experiments were performed at least three times and the results are shown as means ± SD. Differences between mean values were tested for significance using ANOVA. A *P* < 0.05 was considered to be statistically significant.

### Structural Analysis

Comparison of cryo EM structures of AdeB (PDB code 7 kgd) and AdeJ (PDB code 7 m4q) were conducted using the DALI platform for pairwise alignment (Holm, 2020) and the software Coot (Emsley and Cowtan, 2004) and PyMol (Seeliger and de Groot, 2010).

## Results

### Effect of Chlorhexidine Digluconate on *A. baumannii* ATCC 19606

**FIGURE 1:**
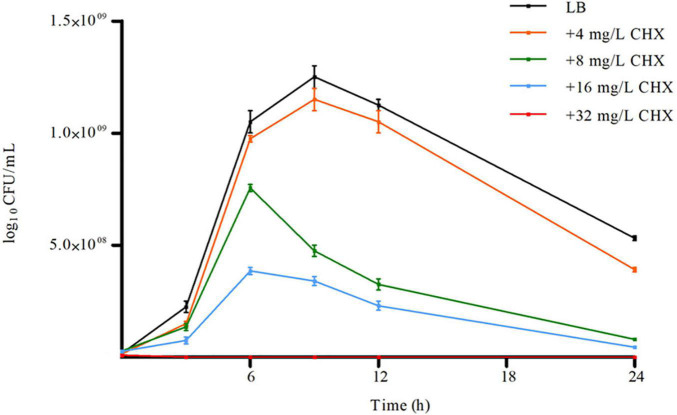
Effect of increasing concentration of CHX on *A. baumannii* ATCC 19606 planktonic growth. Error bars represent standard deviations based on three independent experiments. CFU, colony-forming units.

*A. baumannii* ATCC 19606, AYE, ACICU strains having different antimicrobial susceptibility profiles and classified as susceptible, multidrug-resistant (MDR) and extensively drug-resistant (XDR) as described (Magiorakos et al., 2012), respectively, invariably showed both CHX MIC and MBC values of 32 mg/L and were considered tolerant to CHX (Table 1). Instead, *E. coli* ATCC 25922 showed a CHX MIC/MBC value of 2 mg/L and was considered susceptible (Table 1). *A. baumannii* ATCC 19606 was able to grow and retain viability in the presence of 4–16 mg/L subMIC concentrations of CHX, while *A. baumannii* ATCC 19606 growth was abolished at 32 mg/L CHX (Figure 1). Also, CHX subMIC concentrations of 8 and 16 mg/L decreased stationary phase cell density of *A. baumannii* ATCC 19606 by three and fourfold, respectively (Figure 1).

**TABLE 1:** MIC (mg/L) and MBC (mg/L) values of CHX against *A. baumannii* strains and *E. coli* reference strain.

Strain	CHX		Interpretation
	MIC	MBC	
*A. baumannii* ATCC 19606	32	32	T
*A. baumannii* ACICU	32	32	T
*A. baumannii* AYE	32	64	T
*E. coli* ATCC 25922	2	2	S

*T, tolerant; S, susceptible.*

**FIGURE 2:**
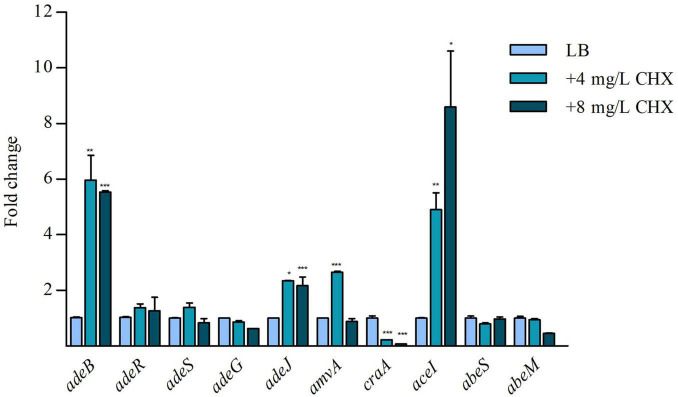
RT-qPCR assay of *adeB, adeR*, *adeS*, *adeG*, *adeJ*, *amvA*, *craA*, *aceI*, *abeS*, and *abeM* expression in the presence of LB and 4 mg/L and 8 mg/L CHX. Relative number of transcripts of each gene was normalized in each condition and calculated using the 2^–^*^ΔΔ^*^ct^ method compared to the expression level in LB control. The mean + standard deviation of relative number of transcripts is shown for each gene. All experiments were performed in triplicate. *p*-values were calculated using ANOVA (*p < 0.05; **p < 0.01; ***p < 0.001).

Because it has been demonstrated that CHX increased the expression of *aceI* efflux pump (EP) gene in *A. baumannii* ATCC 17978 (Hassan et al., 2013), we asked if CHX was able to regulate the expression of EPs genes in ATCC 19606. Preliminary data showed that basal level of expression of *adeB, adeG*, *adeJ*, belonging to RND superfamily, *amvA* and *craA* belonging to MFS superfamily, *aceI*, belonging to PACE superfamily, and *abeS* and *abeM*, belonging to the SMR superfamily were different in *A. baumannii* ATCC 19606. In particular, *aceI*, *adeJ*, *adeB*, and *amvA* were expressed at high levels, with expression levels normalized on *rpoB* of 0.49, 0.34, 0.25, and 0.28, respectively, while *craA*, *abeS*, and *abeM* at low levels (Supplementary Figure 1). As shown in Figure 2, CHX at subMIC concentrations (4 and 8 mg/L) increased the expression of *adeB* and *adeJ* EPs genes by 6x and 2x, respectively, while the expression of *adeG* EP gene and *adeR* and *adeS* regulatory genes were not affected. Moreover, subMIC concentrations of CHX increased the expression of *aceI* EP gene and *amvA* EP gene 5x by 4 mg/mL and 9x by 8 mg/mL, and 2x by 4 mg/mL, respectively (Figure 2). *amvA* EP gene expression was not induced in the presence of 8 mg/mL CHX. On the other hand, subMIC concentrations of CHX decreased the expression of *craA* EP gene 4x by 4 mg/L and 8x by 8 mg/L (Figure 2). The above data indicated that *adeB*, *aceI* and to lesser extent *adeJ* and *amvA* EP genes are activated by CHX in *A. baumannii* ATCC 19606.

### Effect of Efflux Pumps Inactivation on Chlorhexidine Minimum Inhibitory Concentration and Minimum Bactericidal Concentration, Planktonic and Sessile Growth in *A. baumannii* ATCC 19606

To study the molecular mechanisms responsible for tolerance to CHX in *A. baumannii*, we analyzed the effect of inactivation of AdeB and AdeJ, AceI, and AmvA EPs, which are abundantly expressed and positively regulated by CHX in *A. baumannii* ATCC19606, on susceptibility to CHX. To this aim, CHX MIC and MBC were analyzed in *A. baumannii* ATCC 19606 marker-less mutants of *adeB*, *adeJ*, *aceI* and *amvA* EPs genes. As shown in Table 2, CHX MIC and MBC values were decreased by eight and twofold in Δ*adeB* and in Δ*aceI* mutant, respectively, compared with *A. baumannii* ATCC19606; in Δ*amvA* mutant CHX MIC was also decreased by twofold but CHX MBC was not affected. Instead, CHX MIC and MBC in Δ*adeJ* mutant were similar to *A. baumannii* ATCC19606 (Table 2). Furthermore, CHX MIC and MBC values were decreased by 16-fold in Δ*adeB* Δ*aceI* and Δ*adeB* Δ*adeJ* double mutants, eightfold in Δ*amvA* Δ*adeB*, and fourfold in Δ*amvA* Δ*aceI* double mutant, while CHX MIC was decreased by two fold, but CHX MBC not affected in Δ*amvA* Δ*adeJ* and Δ*aceI* Δ*adeJ* double mutants. Moreover, CHX MIC and MBC were decreased by 32-fold in Δ*adeB* Δ*aceI* Δ*adeJ*, 16-fold in Δ*amvA* Δ*adeB* Δ*aceI* and Δ*amvA* Δ*adeB* Δ*adeJ*, and twofold in Δ*amvA* Δ*aceI* Δ*adeJ* triple mutants (Table 2). CHX susceptibility with MIC and MBC values of 2–1 was recovered in Δ*adeB* Δ*aceI* and Δ*adeB* Δ*adeJ* double mutants, and Δ*adeB* Δ*aceI* Δ*adeJ*, Δ*amvA* Δ*adeB* Δ*aceI*, and Δ*amvA* Δ*adeB* Δ*adeJ* triple mutants (Table 2). The above data indicated that CHX MIC and MBC in *A. baumannii* ATCC 19606 were mainly sustained by the expression of *adeB* and that *aceI*, *amvA* and to a lesser extent *adeJ* played an additive effect.

**TABLE 2:** CHX MIC (mg/L) and MBC (mg/L) of *A. baumannii* ATCC 19606 parental strain and EP deletion mutants.

Strain	CHX MIC	
	MIC	MBC
ATCC 19606	32	32
Δ*amvA*	16	32
Δ*aceI*	16	16
Δ*adeB*	4	4
Δ*adeJ*	32	32
Δ*amvA* Δ*aceI*	8	8
Δ*amvA* Δ*adeB*	4	4
Δ*adeB* Δ*aceI*	2	2
Δ*amvA* Δ*adeJ*	16	32
Δ*aceI* Δ*adeJ*	16	32
Δ*adeB* Δ*adeJ*	2	2
Δ*amvA* Δ*adeB* Δ*aceI*	2	2
Δ*adeB* Δ*aceI* Δ*adeJ*	1	1
Δ*amvA* Δ*aceI* Δ*adeJ*	16	16
Δ*amvA* Δ*adeB* Δ*adeJ*	2	2

To further study the role of EPs on CHX susceptibility in *A. baumannii*, we analyzed the effect the EP inhibitor CCCP in *A. baumannii* ATCC 19606 and EPs marker-less mutants. As shown in Table 3, CCCP reduced dose-dependently CHX MIC in *A. baumannii* ATCC 19606 and in Δ*adeJ*, Δ*aceI*, or Δ*amvA* single, double or triple mutants. CCCP reduced CHX MIC in Δ*adeB*, single, double or triple mutants but the effect was not dose-dependent. This indicates that inhibition of efflux pump activity restores susceptibility to CHX in *A. baumannii* ATCC 19606 and in Δ*adeJ*, Δ*aceI*, or Δ*amvA*, but not in Δ*adeB* mutants.

**TABLE 3:** MIC of CHX (mg/L) in combination with CCCP of *A. baumannii* ATCC 19606 parental strain and EP deletion mutants.

Strain	CCCP MIC	CHX MIC			
		CCCP			
		0	0.5	1	2
ATCC 19606	32	32	16	8	8
Δ*amvA*	32	16	8	8	4
Δ*aceI*	32	16	16	8	4
Δ*adeB*	16	4	2	2	2
Δ*adeJ*	32	32	16	16	8
Δ*amvA* Δ*aceI*	16	8	4	4	1
Δ*amvA* Δ*adeB*	32	4	2	2	2
Δ*adeB* Δ*aceI*	16	2	2	2	1
Δ*amvA* Δ*adeJ*	32	16	8	4	1
Δ*aceI* Δ*adeJ*	32	16	16	2	1
Δ*adeB* Δ*adeJ*	8	2	1	1	1
Δ*amvA* Δ*adeB* Δ*aceI*	32	2	1	1	1
Δ*adeB* Δ*aceI* Δ*adeJ*	8	1	1	1	0.5
Δ*amvA* Δ*aceI* Δ*adeJ*	32	16	8	4	1
Δ*amvA* Δ*adeB* Δ*adeJ*	32	2	1	1	1

**FIGURE 3:**
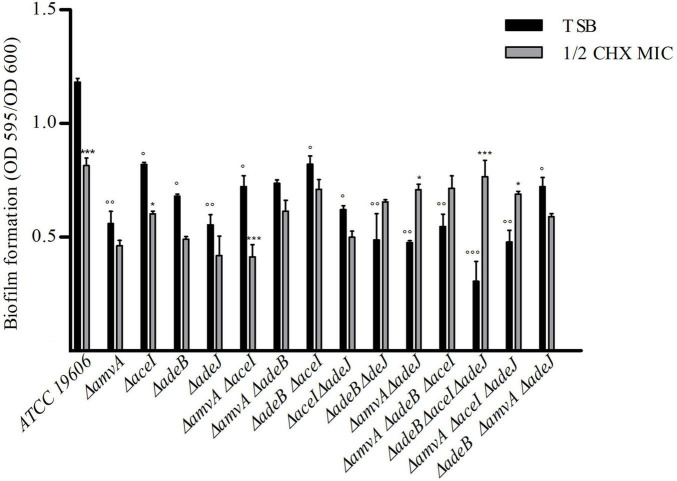
Biofilm formation of A. *baumannii* ATCC 19606 parental strain and single, double and triple deletion mutants in the absence (TSB) or the presence of ½ CHX MIC. *P*-values were calculated using ANOVA (^°^*p* < 0.05, ^°°^*p* < 0.01, or ^°°°^*p* < 0.001 vs. *A. baumannii* ATCC 19606 parental strain; *p < 0.05 or ***p < 0.001 vs. ½ CHX MIC).

We next asked whether EPs knockout gene inactivation might affect the *in vitro* planktonic and sessile growth of *A. baumannii* ATCC 19606. *A. baumannii* ATCC 19606 and single, double or triple Δ*adeJ*, Δ*aceI*, Δ*amvA*, Δ*adeB* mutants showed similar sigmoid growth curves and no difference in growth rates, despite Δ*amvA* Δ*adeB* Δ*aceI* and Δ*amvA* Δ*adeB* Δ*adeJ* triple mutants showed a longer lag phase than *A. baumannii* ATCC 19606 and other deletion mutants (Supplementary Figure 2). We analyzed also biofilm growth of *A. baumannii* ATCC 19606 and single, double or triple EP mutants. As shown in Figure 3, biofilm formation of single, double or triple Δ*adeJ*, Δ*aceI*, Δ*amvA*, Δ*adeB* mutants grown in the absence or in the presence of 1/2 MIC CHX was decreased by 30–50% compared with *A. baumannii* ATCC 19606 parental cells. On the other hand, 1/2 MIC CHX decreased biofilm growth in ATCC 19606 parental, Δ*adeJ*, Δ*aceI*, Δ*amvA*, Δ*adeB* single mutants, Δ*amvA* Δ*aceI*, Δ*amvA* Δ*adeB*, Δ*adeB* Δ*aceI*, and Δ*aceI* Δ*adeJ* double mutants and Δ*amvA* Δ*adeB* Δ*adeJ* triple mutants, while induced biofilm growth in Δ*adeB* Δ*adeJ* or Δ*amvA* Δ*adeJ* double mutants, and Δ*amvA* Δ*adeB* Δ*aceI*, Δ*adeB* Δ*aceI* Δ*adeJ*, or Δ*amvA* Δ*aceI* Δ*adeJ* triple mutants (Figure 3).

### Susceptibility to Benzalkonium Chloride, Dequalinium Chloride, Cetrimide and Triclosan in *A. baumannii* ATCC 19606 Wild Type and Efflux Pump Deletion Mutants

The susceptibility to other biocides, which are used as antiseptics or disinfectants (McDonnell and Russell, 1999), was analyzed in *A. baumannii* ATCC 19606 wild type and EP deletion mutants. In accordance with previous findings (Chen et al., 2009), *A. baumannii* ATCC19606 and single EP deletion mutants showed TRI MIC and MBC of 0.06 and 0.125 mg/L, respectively, and were considered susceptible to TRI (Supplementary Table 3). On the contrary, *A. baumannii* ATCC19606 and single EP deletion mutants were tolerant to quaternary ammonium compounds DQ and CT, showing MIC and MBC values of 32–256 and 16–64 mg/L, respectively (Supplementary Table 3).

The mechanisms responsible for tolerance to BZK was studied in detail in *A. baumannii* ATCC 19606 parental strain and marker-less mutants of *adeB*, *adeJ*, *aceI* and *amvA* EPs genes. As shown in Table 4, BZK MIC and MBC values were decreased by four, two, and onefold in Δ*adeB*, Δ*amvA*, and Δ*aceI* mutants, respectively, compared with *A. baumannii* ATCC19606; BZK MIC and MBC were not affected in Δ*adeJ* mutant. Also, BZK MIC and MBC values were decreased by eightfold in Δ*amvA* Δ*adeB*, Δ*adeB* Δ*aceI*, and Δ*adeB* Δ*adeJ* double mutants, and twofold in Δ*amvA* Δ*aceI*, Δ*amvA* Δ*adeJ*, and Δ*aceI* Δ*adeJ* double mutants. Moreover, BZK MIC and MBC were decreased by 16-fold in Δ*adeB* Δ*aceI* Δ*adeJ* and Δ*amvA* Δ*adeB* Δ*adeJ*, eightfold in Δ*amvA* Δ*adeB* Δ*aceI*, fourfold in Δ*amvA* Δ*aceI* Δ*adeJ* triple mutants (Table 4). BZK susceptibility with MIC and MBC values of 2 was recovered in Δ*adeB* Δ*aceI* Δ*adeJ* and Δ*amvA* Δ*adeB* Δ*adeJ* triple mutants (Table 4). The above data indicated that BZK MIC and MBC in *A. baumannii* ATCC 19606 were mainly regulated by the functioning of *adeB* and to a lesser extent *amvA, aceI*, and *adeJ* EPs.

**TABLE 4:** BZK MIC (mg/L) and MBC (mg/L) of *A. baumannii* ATCC 19606 parental strain and EP deletion mutants.

Strain	BZK	
	MIC	MBC
ATCC 19606	32	32
Δ*amvA*	16	16
Δ*aceI*	16	16
Δ*adeB*	8	8
Δ*adeJ*	32	32
Δ*amvA* Δ*aceI*	16	16
Δ*amvA* Δ*adeB*	4	4
Δ*adeB* Δ*aceI*	4	4
Δ*amvA* Δ*adeJ*	16	16
Δ*aceI* Δ*adeJ*	16	16
Δ*adeB* Δ*adeJ*	4	4
Δ*amvA* Δ*adeB* Δ*aceI*	4	8
Δ*adeB* Δ*aceI* Δ*adeJ*	2	2
Δ*amvA* Δ*aceI* Δ*adeJ*	8	8
Δ*amvA* Δ*adeB* Δ*adeJ*	2	2

*BZK, Benzalkonium chloride.*

### Structural Comparison of AdeB and AdeJ Protomers

**FIGURE 4:**
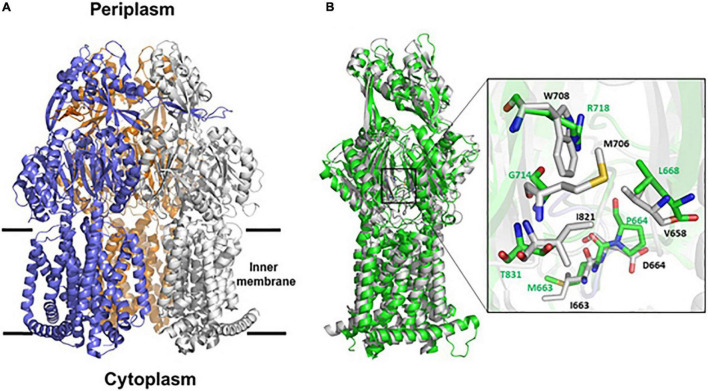
Structural representation of AdeB and AdeJ pumps of *A. baumannii*. **(A)** Cartoon representation of AdeB heterotrimer (pdb code 7 kgd); the three protomers are represented in blue, white and orange. **(B)** Superposition between the structures of AdeB and AdeJ (pdb code 7m4q) protomers. The two structures superpose with a backbone root mean square deviations (rmsd) of 2.5, 2.9, 3.0 Å on chains A, B, and C, respectively. The inset shows a zoom of the entrance sites of AdeB (white) and AdeJ (green). AdeB residues are labeled black whereas corresponding AdeJ residues are labeled green.

Overall, AdeB and AdeJ are two highly homologous proteins sharing a sequence identity of 49%. Both AdeB and AdeJ adopt a homotrimeric structure, with the typical RND-like fold (Su et al., 2019; Morgan et al., 2021; Zhang et al., 2021). Similar to AcrB of *E. coli* (seqid 50%), they are composed of a transmembrane domain formed by 12 transmembrane (TM) helices and a large periplasmic domain (Figure 4A). In this structural organization, the periplasmic domain harbors an entrance, a proximal and a distal substrate binding pockets (PBP and DBP, respectively). The PBP is separated from the DPB by a so-called “gate-loop” (or G-loop). Another conserved flexible loop (F-loop) connects the cleft entrance to the proximal drug-binding pocket. These loops are crucial to substrate discrimination in AcrB (Schuster et al., 2016). During substrate extrusion, AdeB and AcrB are thought to pass through a conformational change that forces the substrate to move from the PBP to the DBP for final extrusion (Schuster et al., 2016; Morgan et al., 2021).

A structural comparison of AdeB and AdeJ protomers was performed to analyze whether differences in the structural features of the two pumps may account for the major role observed for AdeB, compared to AdeJ, on CHX extrusion and susceptibility. As shown in Figure 4B, AdeB and AdeJ share a strictly conserved fold, with root mean square deviations (rmsd) ranging between 2.5 and 3.0 Å on the three chains. The analysis of the entrance binding sites of AdeB and AdeJ suggests different features that may explain a different involvement in CHX transport. Most relevant, the conserved W708 of AdeB is replaced by an arginine residue (R718) in AdeJ (Figure 4B). Other residues belonging to this cavity also differ. Specifically, V658, M706, I861 are replaced by L668, G714, and T831, respectively. These differences in the composition of the entrance site of AdeJ, compared to AdeB, make the pocked positively charged and not prone to bind the positively charged CHX. Significant differences are also observed in the F loops of the two pumps. In AdeB, the F-loop (661-PAIDELGT-668) resembles that of AcrB (669-PAIVELGT-676) of *E. coli*, in which residue I671 has been shown to be important for drug discrimination (Schuster et al., 2016). Differently, the F-loop of AdeJ does not contain this key isoleucine (669-PAMPELGV-676), which is thought to be part of a preferential small-drug entrance pathway. Additionally, a more negatively charged F-loop (due to the charge contribution of D664) in AdeB may also contribute to its stronger involvement in the transport of the positively charged CHX.

### Effect of Piperine and Resveratrol on Chlorhexidine and Benzalkonium Susceptibility and Expression of Efflux Pumps Genes in *A. baumannii* ATCC 19606 Wild Type and Deletion Mutants

We next screened two natural compounds, RV and PIP, which have shown promising activity as EPs inhibitors (Sharma et al., 2010; Mirza et al., 2011; Singkham-In et al., 2020). We tested if these non-toxic compounds can decrease CHX MIC in *A. baumannii* ATCC 19606 and EPs gene knockout mutants and restore susceptibility to CHX. Both PIP and RV showed no antimicrobial activity against *A. baumannii* ATCC 19606 and Δ*adeJ*, Δ*aceI*, Δ*amvA*, Δ*adeB* mutants (MIC > 1,024 mg/L) (Tables 5, 6). We then determined the antimicrobial activity of PIP in combination with CHX by *in vitro* combination assay. As shown in Table 5, increasing doses of PIP up to 128 mg/L decreased CHX MIC and MBC by four fold in *A. baumannii* ATCC 19606 and by two to eightfold in Δ*adeJ*, Δ*aceI*, Δ*amvA*, Δ*adeB* mutants, being able to restore CHX susceptibility in single, double and triple mutants with inactivation of *adeB* gene. Furthermore, RV from 32 to 128 mg/L decreased dose-dependently CHX MIC and MBC and restored CHX susceptibility in *A. baumannii* ATCC 19606 and Δ*adeJ*, Δ*aceI*, Δ*amvA*, Δ*adeB* single, double and triple mutants. In particular, CHX susceptibility was restored by RV at 128 mg/L in *A. baumannii* ATCC 19606 and Δ*aceI*, Δ*amvA*, Δ*adeB*, or Δ*adeJ* single mutants, 64 mg/L in all double or EP triple mutants, 32 mg/L in double or triple EP mutants harboring deletion of *adeB* (Table 6).

**TABLE 5:** MIC (mg/L) and MBC of CHX (mg/L) in combination with PIP in *A. baumannii* ATCC 19606 parental strain and EP deletion mutants.

Strain	PIP MIC	CHX MIC (MBC)					
		PIP					
		0	8	16	32	64	128
ATCC 19606	>1,024	32 (32)	32 (32)	16 (16)	8 (8)	8 (8)	8 (8)
Δ*amvA*	>1,024	16 (16)	16 (16)	8 (16)	8 (8)	8 (8)	8 (8)
Δ*aceI*	>1,024	16 (16)	16 (16)	16 (16)	8 (16)	8 (16)	8 (8)
Δ*adeB*	>1,024	4 (4)	4 (4)	2 (4)	2 (4)	2 (2)	2 (2)
Δ*adeJ*	>1,024	32 (32)	32 (32)	16 (16)	16 (16)	8 (8)	4 (4)
Δ*amvA* Δ*aceI*	>1,024	8 (8)	8 (8)	8 (8)	4 (8)	4 (4)	4 (4)
Δ*amvA* Δ*adeB*	>1,024	4 (4)	4 (4)	1 (4)	1 (2)	1 (2)	1 (2)
Δ*adeB* Δ*aceI*	>1,024	2 (2)	2 (2)	1 (1)	1 (1)	1 (1)	1 (1)
Δ*amvA* Δ*adeJ*	>1,024	16 (16)	16 (16)	16 (16)	8 (16)	8 (8)	8 (8)
Δ*adeB* Δ*adeJ*	>1,024	2 (2)	2 (2)	1 (2)	1 (2)	1 (1)	1 (1)
Δ*aceI* Δ*adeJ*	>1,024	16 (16)	16 (16)	16 (16)	8 (16)	8 (8)	8 (8)
Δ*amvA* Δ*adeB* Δ*aceI*	>1,024	2 (2)	2 (2)	0.5 (2)	0.5 (1)	0.5 (1)	0.5 (1)
Δ*adeB* Δ*aceI* Δ*adeJ*	>1,024	1 (1)	1 (1)	1 (1)	0.5 (0.5)	0.5 (0.5)	0.5 (0.5)
Δ*amvA* Δ*aceI* Δ*adeJ*	>1,024	16 (16)	16 (16)	8 (8)	4 (8)	4 (4)	4 (4)
Δ*amvA* Δ*adeB* Δ*adeJ*	>1,024	2 (2)	2 (2)	1 (2)	0.5 (1)	0.5 (0.5)	0.5 (0.5)

**TABLE 6:** RV effect on CHX MIC (mg/L) and CHX MBC (mg/L) in *A. baumannii* ATCC 19606 parental strain and EP deletion mutants.

Strain	RV MIC	CHX MIC (MBC)			
		RV			
		0	32	64	128
ATCC 19606	>1,024	32 (32)	8 (16)	4 (8)	<0.5 (2)
Δ*amvA*	>1,024	16 (16)	8 (8)	4 (8)	<0.5 (2)
Δ*aceI*	>1,024	16 (16)	8 (16)	4 (8)	<0.5 (2)
Δ*adeB*	>1,024	4 (4)	4 (4)	4 (4)	<0.5 (2)
Δ*adeJ*	>1,024	32 (32)	8 (16)	4 (16)	<0.5 (2)
Δ*amvA* Δ*aceI*	>1,024	8 (8)	4 (4)	1 (4)	<0.5 (1)
Δ*amvA* Δ*adeB*	>1,024	4 (4)	2 (4)	1 (2)	<0.5 (2)
Δ*adeB* Δ*aceI*	>1,024	2 (2)	2 (2)	1 (2)	<0.5 (1)
Δ*amvA* Δ*adeJ*	>1,024	16 (16)	4 (4)	1 (2)	<0.5 (1)
Δ*adeB* Δ*adeJ*	>1,024	2 (2)	1 (2)	<0.5 (1)	<0.5 (0.5)
Δ*aceI* Δ*adeJ*	>1,024	16 (16)	4 (8)	1 (2)	<0.5 (0.5)
Δ*amvA* Δ*adeB* Δ*aceI*	>1,024	2 (2)	0.5 (1)	0.5 (1)	<0.5 (0.5)
Δ*adeB* Δ*aceI* Δ*adeJ*	>1,024	1 (1)	0.5 (1)	<0.5 (1)	<0.5 (0.5)
Δ*amvA* Δ*aceI* Δ*adeJ*	>1,024	16 (16)	4 (8)	0.5 (1)	<0.5 (0.5)
Δ*amvA* Δ*adeB* Δ*adeJ*	>1,024	2 (2)	1 (1)	<0.5 (0.5)	<0.5 (0.5)

**FIGURE 5:**
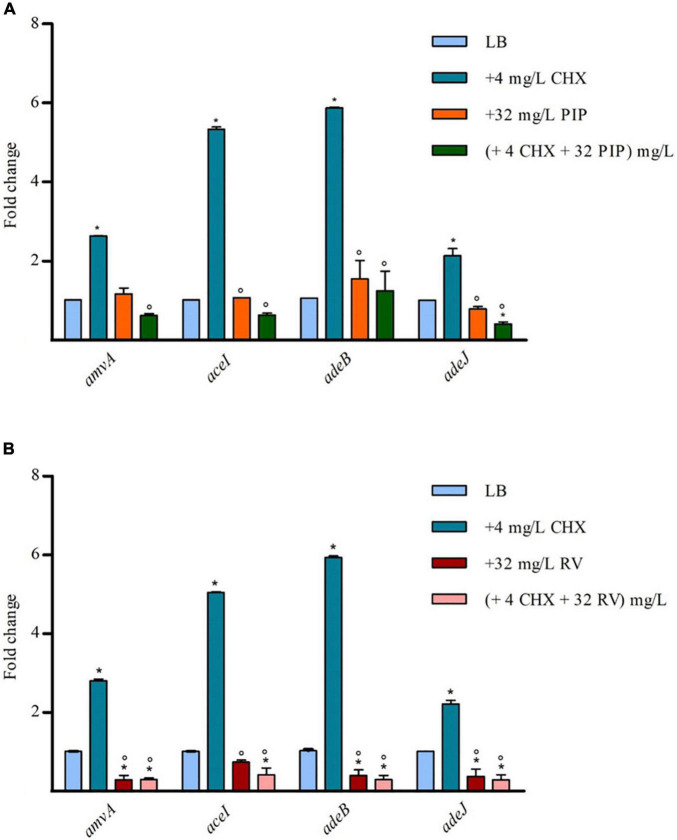
RT-qPCR assay of *amvA*, *aceI*, *adeB*, and *adeJ* genes expression in the absence (LB) or presence of 4 mg/L CHX alone or in combination with 32 mg/L PIP **(A)** or 32 mg/L RV **(B)**. Relative number of transcripts of each gene was normalized in each condition and calculated using the 2^–^*^ΔΔ^*^ct^ method compared to the expression level in LB control. The mean ± standard deviation of relative number of transcripts is shown for each gene. All experiments were performed in triplicate. *P*-values were calculated using ANOVA (*p < 0.01 vs. LB;°*p* < 0.01 vs. 4 mg/L CHX).

To assess whether the effect of PIP and RV on CHX susceptibility was mediated by inhibition of EPs expression, we analyzed *amvA*, *aceI*, *adeB*, and *adeJ* expression in *A. baumannii* ATCC 19606 in the presence of 4 mg/L subMIC CHX in combination with 32 mg/L PIP or 32 mg/L RV. As shown in Figure 5A, PIP counteracted CHX-dependent increased expression of *amvA*, *aceI*, *adeB*, and *adeJ*, while it did not affect basal EP gene expression. On the other hand, resveratrol inhibited both basal and CHX-dependent increased expression of *amvA*, *aceI*, *adeB*, and *adeJ*, the highest effect found for *adeB* and *amvA* (Figure 5B). The above data suggested that different effects of PIP and RV on CHX MIC in *A. baumannii* ATCC 19606 were mediated by distinct regulation of *amvA*, *aceI*, *adeB*, and *adeJ* expression.

The effect of RV was also analyzed on BZK MIC and MBC in *A. baumannii* ATCC 19606 parental strain and EP deletion mutants. As shown in Table 7, RV from 32 mg/L to 128 mg/L decreased dose-dependently BZK MIC and MBC and restored BZK susceptibility in *A. baumannii* ATCC 19606 and single, double and triple EP deletion mutants. BZK susceptibility was restored by RV at 128 mg/L in *A. baumannii* ATCC 19606 and Δ*aceI* or Δ*amvA*, single mutants, 64 mg/L in Δ*adeB*, or Δ*adeJ* single mutants and in all double or EP triple mutants, 32 mg/L in Δ*adeB* single mutant and in all, but not Δ*amvA* Δ*adeJ*, double mutants (Table 7).

**TABLE 7:** RV effect on BZK MIC (mg/L) and MBC (mg/L) in *A. baumannii* ATCC 19606 parental strain and EP deletion mutants.

Strain	BZK MIC (MBC)			
	RV			
	0	32	64	128
ATCC 19606	32 (32)	16 (32)	4 (16)	0.5 (1)
Δ*amvA*	16 (16)	8 (8)	4 (4)	<0.5 (0.5)
Δ*aceI*	16 (16)	16 (16)	4 (4)	<0.5 (0.5)
Δ*adeB*	8 (8)	2 (4)	1 (1)	<0.5 (0.5)
Δ*adeJ*	32 (32)	8 (16)	2 (4)	<0.5 (1)
Δ*amvA* Δ*aceI*	16 (16)	2 (2)	1 (1)	<0.5 (0.5)
Δ*amvA* Δ*adeB*	4 (4)	1 (2)	0.5 (0.5)	<0.5 (0.5)
Δ*adeB* Δ*aceI*	4 (4)	2 (2)	1 (2)	<0.5 (0.5)
Δ*amvA* Δ*adeJ*	16 (16)	4 (4)	1 (1)	<0.5 (0.5)
Δ*adeB* Δ*adeJ*	4 (4)	1 (1)	0.5 (0.5)	<0.5 (0.5)
Δ*aceI* Δ*adeJ*	16 (16)	2 (2)	1 (1)	<0.5 (0.5)
Δ*amvA* Δ*adeB* Δ*aceI*	4 (8)	0.5 (1)	0.5 (1)	<0.5 (0.5)
Δ*adeB* Δ*aceI* Δ*adeJ*	2 (2)	0.5 (0.5)	0.5 (0.5)	<0.5 (0.5)
Δ*amvA* Δ*aceI* Δ*adeJ*	8 (8)	2 (16)	1 (2)	<0.5 (0.5)
Δ*amvA* Δ*adeB* Δ*adeJ*	2 (2)	0.25 (1)	<0.5 (0.5)	<0.5 (0.5)

**FIGURE 6:**
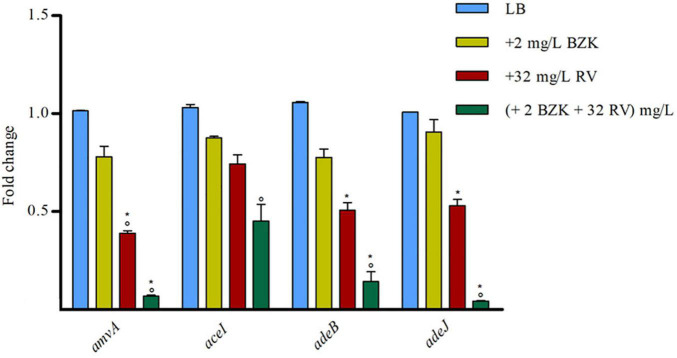
RT-qPCR assay of *amvA*, *aceI*, *adeB*, and *adeJ* genes expression in the absence (LB) or presence of 2 mg/L BZK alone or in combination with 32 mg/L RV. Relative number of transcripts of each gene was normalized in each condition and calculated using the 2^–^*^ΔΔ^*^ct^ method compared to the expression level in LB control. The mean ± standard deviation of relative number of transcripts is shown for each gene. All experiments were performed in triplicate. *P*-values were calculated using ANOVA (*p < 0.01 vs. LB; °*p* < 0.01 vs. 2 mg/L BZK).

We analyzed also the effect of 2 mg/L BZK alone or in combination with 32 mg/L RV on *amvA*, *aceI*, *adeB*, and *adeJ* expression. As shown in Figure 6, two mg/L BZK alone inhibited in a non-significant way EP gene expression, and 2 mg/L BZK in combination with 32 mg/L RV significantly inhibited *amvA*, *adeB*, and *adeJ* expression by 10–15-fold and *aceI* expression by twofold respect to untreated cells. The above data indicated that the effect of RV on BZK susceptibility was mediated by inhibition of *amvA*, *adeB*, *adeJ*, and to a lesser extent *aceI* expression.

## Discussion

The present study analyzes the molecular mechanism responsible for adaptation and tolerance of *A. baumannii* to CHX and BZK. Our data demonstrate that *adeB*, *aceI* and to lesser extent *adeJ* and *amvA* EP genes are activated by CHX in *A. baumannii* ATCC 19606 and that inactivation of EP genes decreases CHX MIC and MBC and restores CHX susceptibility in *A. baumannii* ATCC 19606. We show that subMIC concentrations of CHX enhance the expression of *aceI* efflux pump gene five to nine-fold, whereas that of *adeB* is enhanced sixfold. Despite this observation, CHX MIC and MBC decrease is significantly higher (eightfold) in Δ*adeB* compared to Δ*amvA* or Δ*aceI* mutant (two fold), or Δ*adeJ* mutant (no decrease). Single, double and triple mutants with inactivation of *adeB* gene showed an additive effect on CHX MIC and MBC (Table 2). Our data are in agreement with and extend previous studies showing that resistance to CHX in *A. baumannii* ATCC 17978 is dependent on increased expression of *aceI* in *A. baumannii* ATCC17978 (Hassan et al., 2013) and that inactivation of AceI EP (Hassan et al., 2013), AdeB or AdeJ RND EPs (Rajamohan et al., 2010a) or AmvA MFS EP (Rajamohan et al., 2010b), AceI or AdeB (Tucker et al., 2014) restores susceptibility to CHX and other disinfectants in *A. baumannii*. In accordance with previous findings (Tucker et al., 2014), data reported herein suggest a major involvement of AdeB in CHX transport compared to AceI. Susceptibility to CHX suggests an even lower involvement of the other pumps (AdeJ, AmvA) in CHX efflux, with no effect on MIC nor on MBC observed upon Δ*adeJ* mutation. In accordance with previous study (Yoon et al., 2015), we showed that inactivation of either AmvA, AceI, AdeB, or AdeJ alone or in combination did not affect planktonic growth but reduced biofilm formation by 30–50% in the absence and in the presence of 1/2 MIC CHX. However, subMIC CHX concentrations increase biofilm formation in Δ*amvA* Δ*adeJ*, Δ*adeB* Δ*aceI* Δ*adeJ*, and Δ*amvA* Δ*aceI* Δ*adeJ* mutants compared to untreated cells, thus suggesting that CHX positively regulate the phenomenon. Overall, our data indicates that EPs have pleiotropic effect and regulate multiple functions in addition to tolerance to disinfectants (Yoon et al., 2015; Du et al., 2018; Kornelsen and Kumar, 2021).

Our data demonstrate that tolerance to BZK in *A. baumannii* ATCC 19606 is regulated by AdeB EP and that AmvA, AceI and AdeJ EPs play a role also. BZK MIC was decreased by fourfold in Δ*adeB* mutant, and twofold in Δ*amvA* and Δ*aceI* mutants, respectively; EPs double and triple deletion mutants showed an additive effect on BZK MIC (Table 4). BZK susceptibility is recovered in Δ*adeB* Δ*aceI* Δ*adeJ* and Δ*amvA* Δ*adeB* Δ*adeJ* triple mutants. This is in partial agreement with previous study showing that inactivation of AmvA MFS EP decreases BZK MIC by fourfold in *A. baumannii* but not restores full susceptibility to biocide (Rajamohan et al., 2010b). In keeping with this, the data shown herein demonstrate that simultaneous inactivation of AdeB, AmvA, and AdeJ or AceI is necessary to restore BZK susceptibility in *A. baumannii*.

Importantly, AdeB and AdeJ are two highly homologous proteins sharing a sequence identity of 49%. Both *adeB* and *adeJ* genes are abundantly expressed at basal level, showing normalized expression level of 0.25 and 0.34, respectively (Supplementary Figure 1), but *adeB* is 3x higher expressed than *adeJ* in the presence of CHX (Figure 2). Also, *A. baumannii* ATCC 19606 does not possess the *adeC* gene of the *adeABC* operon and may use an alternate outer membrane protein (OMP), likely AdeK, of the constitutive efflux pump, AdeIJK, as described in other *A. baumannii* strains (Sugawara and Nikaido, 2014). However, we observe a completely different involvement of the two RND-type efflux pumps in CHX extrusion and tolerance, with AdeABC playing a central role and AdeIJK being only marginal in this mechanism (Table 2) and we postulate that differences in the structure between AdeB and AdeJ protomers may be responsible for this. The structural comparison of AdeB and AdeJ shows different features at the entrance binding site, such as W708, V658, M706, I861 in AdeB, which are replaced by R718, L668, G714, and T831 in AdeJ, respectively. Overall, a more positive electrostatic potential surface at the entrance site of AdeJ, due to R817, may render this pump not prone to bind the positively charged CHX. Additionally, the F-loop of AdeB presents different features than that of AdeJ, as it is more negatively charged (due to the charge contribution of D664) and contains a key isoleucine residue, I671, which was shown to be important in AcrB (Schuster et al., 2016). These features may contribute to its stronger involvement in the transport of the positively charged CHX by AdeB (Figure 4B). Future experimental data will be necessary to validate the impact of specific residues in AdeB protomer on CHX efflux in *A. baumannii*.

In this work, we also searched for EP inhibitors that restore CHX susceptibility, to tackle *A. baumannii* tolerance to CHX and BZK induced by EP pumps. As a first compound, CCCP showed a significant effect on CHX MIC (Table 3). However, due to the toxicity of this compound, we analyzed the effects on CHX susceptibility of two antioxidant molecules, the non-toxic PIP and RV. As a result, both PIP and RV were able to decrease CHX MIC and MBC in *A. baumannii* ATCC 19606 and EP deletion mutants. In particular, PIP was able to restore CHX susceptibility only in single, double and triple mutants with inactivation of *adeB* gene. In partial agreement with our data, PIP inhibited rifampicin-induced expression of Rv1258c multidrug efflux pump and rifampicin MIC in *Mycobacterium tuberculosis* (Sharma et al., 2010). Similarly, PIP has been demonstrated to inhibit ethidium bromide efflux and mupirocin resistance in methicillin-resistant *S. aureus* (Mirza et al., 2011). Our data also demonstrated that RV has higher efficacy than PIP on CHX susceptibility, being resveratrol able to restore CHX susceptibility dose-dependently both in *A. baumannii* ATCC 19606 and in EP deletion mutants. Coherent with this finding, we show that PIP inhibits CHX-induced, though not basal, expression of EP genes. In addition, consistent with previous data (Singkham-In et al., 2020) we find that RV is able to inhibit both basal levels and CHX-induced expression of *amvA*, *aceI*, *adeB*, and *adeJ* genes in *A. baumannii* ATCC 19606. The differential effects of PIP and RV on CHX MIC is likely to be ascribed to their different ability to inhibit EPs gene expression.

Our data also demonstrated that RV restored BZK susceptibility both in *A. baumannii* ATCC 19606 and in EP deletion mutants. Although unlike CHX, BZK does not induce the expression of EPs genes, RV alone or in the presence of BZK inhibited *amvA*, *aceI*, *adeB* and *adeJ* expression, the effect of RV and BZK being synergic for *amvA*, *adeB*, *adeJ*. Based on this, we hypothesize that the effect of RV on BZK susceptibility in *A. baumannii* is mediated by the inhibition of expression of EPs.

## Conclusion

The data reported in this study demonstrate that tolerance to CHX and BZK in *A. baumannii* is mediated by the activation of EPs. In particular, *adeB*, *adeJ*, *aceI*, and *amvA* expression is induced by CHX; EPs gene inactivation inhibits both CHX and BZK MIC in an additive manner, with AdeB EP playing a major role. We also identified PIP and RV as non-toxic compounds able to inhibit EPs gene expression and CHX or BZK tolerance in *A. baumannii*. Our data demonstrate that co-treatments of RV and CHX or RV and BZK restore susceptibility to biocides in *A. baumannii.*

*A. baumannii* ATCC19606 and EP inactivation mutants described herein may represent a useful model system to study the molecular mechanisms responsible for tolerance to biocides other than CHX and BZK in *A. baumannii* and to identify innovative molecules and combination regimens, which are able to restore susceptibility to disinfectants in *A. baumannii*. The combination of RV may represent a useful strategy to maintain susceptibility to biocides in *A. baumannii* and other nosocomial pathogens.

## Data Availability Statement

The original contributions presented in the study are included in the article/Supplementary Material, further inquiries can be directed to the corresponding author/s.

## Author Contributions

ED and RZ conceived the study and participated in its design and coordination. AM, EE, and MB performed laboratory experiments. RB, MT, ED, and RZ performed data analyses. AM, EE, RB, ED, and RZ wrote the manuscript. All authors read and approved the final manuscript.

## Conflict of Interest

The authors declare that the research was conducted in the absence of any commercial or financial relationships that could be construed as a potential conflict of interest.

## Publisher’s Note

All claims expressed in this article are solely those of the authors and do not necessarily represent those of their affiliated organizations, or those of the publisher, the editors and the reviewers. Any product that may be evaluated in this article, or claim that may be made by its manufacturer, is not guaranteed or endorsed by the publisher.
