# Supplementary figures and images for: Inhibition of AdeB, AceI, and AmvA Efflux Pumps Restores Chlorhexidine and Benzalkonium Susceptibility in Acinetobacter baumannii ATCC 19606
Source: Front Microbiol. 2022 Feb 7;12:790263. doi: 10.3389/fmicb.2021.790263 (PMC8859242; doi:10.3389/fmicb.2021.790263)

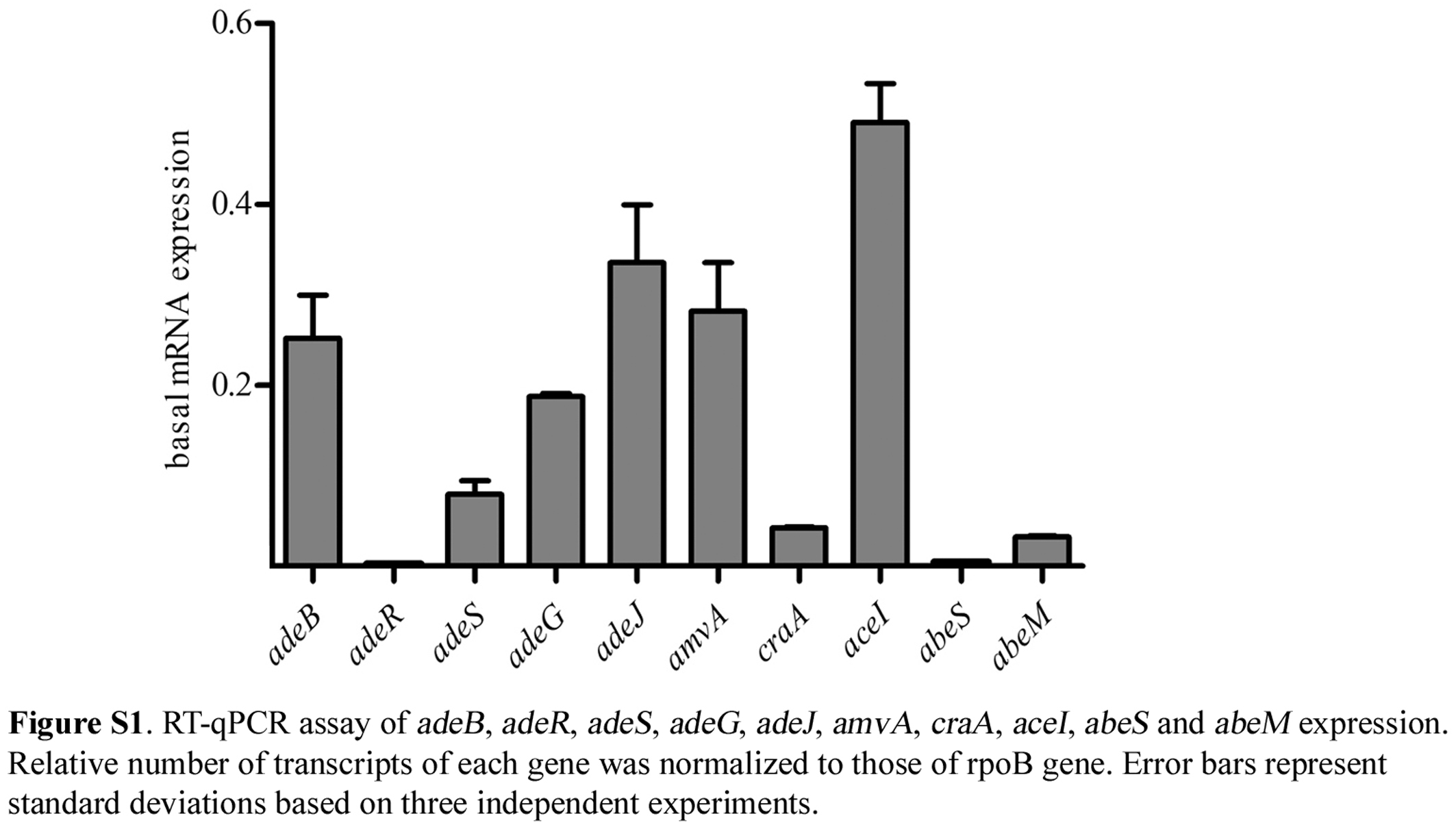

Supplement: Supplementary file 1 [file Image_1.JPEG]

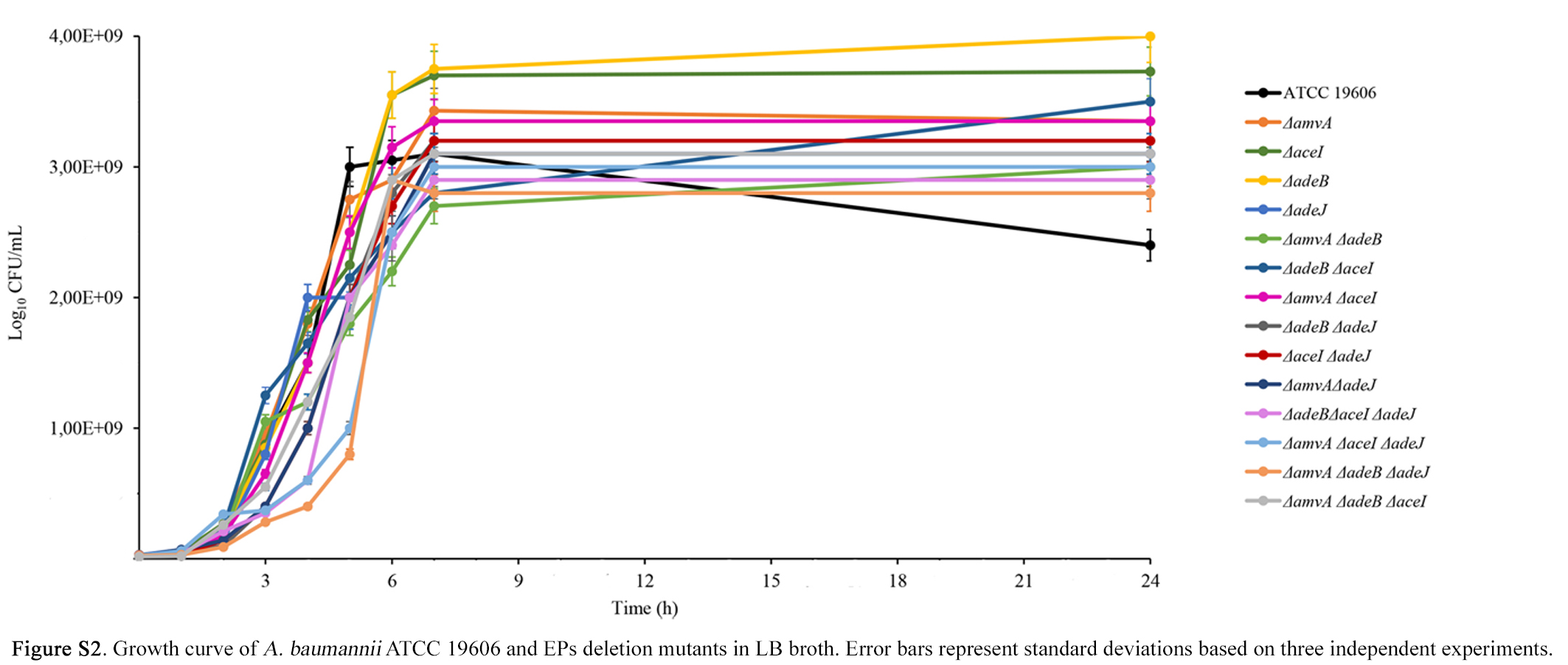

Supplement: Supplementary file 2 [file Image_2.JPEG]
