# Supplementary material for: Inhibition of AdeB, AceI, and AmvA Efflux Pumps Restores Chlorhexidine and Benzalkonium Susceptibility in Acinetobacter baumannii ATCC 19606
Source: Front Microbiol. 2022 Feb 7;12:790263. doi: 10.3389/fmicb.2021.790263 (PMC8859242; doi:10.3389/fmicb.2021.790263)
Supplement: Supplementary file 3 [file Data_Sheet_1.PDF]

Table S1. Oligonucleotides for gene deletion experiments.

Gene deletion	*amvA*-UP	Fw: tatGCGGCCGCGGCGAATAATTGCTTCGGTA (*NotI* site in red)	712	This study
		Rv: gctGGATCCCATAACGCTCTTCAATATCCAAAAC (*BamHI* site in red)		
	amvA-DW	Fw: tatGGATCCTTGTCTGGTTTAGTTTTCCGAAG (*BamHI* site in red)	704	
		Rv: tatGCATGCTTTGTGGGTCTGTCGTAGCA (*SphI* site in red)		
	adeB-UP	Fw: tatGCGGCCGCAGGGCGATACCAATACGATG (*NotI* site in red)	683	
		Rv: gctGGATCCGCGATAACCCAAGCAAAAAC (*BamHI* site in red)		
	adeB-DW	Fw: tatGGATCCTGATGTTAGTTGTGCCACTCG (*BamHI* site in red)	477	
		Rv: tatGCATGCGAGTATTTCCCCTGATGCATTG (*SphI* site in red)		
	adeJ-UP	Fw: tatGCGGCCGCAGCGTTTGTTCTGCCAGTCT (*NotI* site in red)	734	
		Rv: gctGGATCCTTGGTGTACTTGGTGGCGTA (*BamHI* site in red)		
	adeJ-DW	Fw: tatGGATCCTACCTGATTGAGCCGGTTTC (*BamHI* site in red)	446	
		Rv: tatGCATGCAGGGCAACTTGCTTTCTCTG (*SphI* site in red)		
	aceI- UP	Fw: tatGCGGCCGCAAGCAAGCAGAGTGGTGGTT (*NotI* site in red)	816	
		Rv: gctGGATCCGCATGAATGAGTCTTCTCTTGG (*BamHI* site in red)		
	aceI- DW	Fw: tatGGATCCGCAGCATCACTTCACTAATTCAC (*BamHI* site in red)	763	
		Rv: tatGCATGCTGAGCGTGGTTTCTTTGATG (*SphI* site in red)		

1. Michael Hornsey, 2010 JAC doi:10.1093/jac/dkq218
2. Paul G. Higgins, 2004 JAC doi:10.1093/jac/dkh427
3. Machado D, J Med Microbiol. 2018 doi: 10.1099/jmm.0.000741
