# Supplementary material for: Inhibition of AdeB, AceI, and AmvA Efflux Pumps Restores Chlorhexidine and Benzalkonium Susceptibility in Acinetobacter baumannii ATCC 19606
Source: Front Microbiol. 2022 Feb 7;12:790263. doi: 10.3389/fmicb.2021.790263 (PMC8859242; doi:10.3389/fmicb.2021.790263)
Supplement: Supplementary file 4 [file Data_Sheet_2.PDF]

Table S2. Oligonucleotides for RT-PCR experiments.

	Primer	Sequence (5'-3')	Amplicon size (bp)	Reference
**RT-PCR**	*rpoB*	Fw: GAGTCTAATGGCGGTGGTTC	110	1
		Rv: ATTGCTTCATCTGCTGGTTG		
	adeB	Fw: GGATTATGGCGACAGAAGGA	106	2
		Rv: AATACTGCCGCCAATACCAG		
	adeS	Fw: GCCGTCGTATTGAGCAAGTT	158	This study
		Rv: ATCGTCTTGGAACTCGGTTG		
	adeR	Fw: ACGCCAAAAAGCTCAGACTC	143	This study
		Rv: CAGTGATTCATAAGTTCTCCGC		
	adeG	Fw: ACTGCAACTTGAAGACCGA	191	3
		Rv: CATCTGTAACAGCAACGCC		
	adeJ	Fw: CTTCCAGCAATGCCTGAAC	153	3
		Rv: ATTTGGACGCACACCTACA		
	amvA	Fw: CACTGATTATGGCGGGTTTG	163	This study
		Rv: CCTAAGACAGCACGAGAAGC		
	craA	Fw: AGGCCAACGTTAACCCAA	175	3
		Rv: TCATGGCACTGGGGTTTC		
	aceI	Fw: GTGTTTATGGCAGTGGTTTCA	148	This study
		Rv: CAGTCGCAATCAGCAAACCA		
	abeS	Fw: GCAACTTCAGCATTAAAAGCAT	159	3
		Rv: AATAATACCTGCGCCTGACC		
	abeM	Fw: TAAGGCTTCGGCTTATCGAA	190	This study
		Rv: TACTTGGTGTGCGGCAATAA		
