# Supplementary material for: Inhibition of AdeB, AceI, and AmvA Efflux Pumps Restores Chlorhexidine and Benzalkonium Susceptibility in Acinetobacter baumannii ATCC 19606
Source: Front Microbiol. 2022 Feb 7;12:790263. doi: 10.3389/fmicb.2021.790263 (PMC8859242; doi:10.3389/fmicb.2021.790263)
Supplement: Supplementary file 5 [file Data_Sheet_3.PDF]

Table S3. MIC (mg/L) and MBC (mg/L) values of dequalinium chloride (DQ), cetrimide (CT) and triclosan (TRI) against *A. baumannii* strain ATCC 19606 parental strain and EP deletion mutants.

**Strain**	**DQ**		**CT**		**TRI**	
	**MIC**	**MBC**	**MIC**	**MBC**	**MIC**	**MBC**
ATCC 19606	64	256	32	64	0.06	0.125
Δ*amvA*	32	64	32	32	0.06	0.125
Δ*aceI*	64	128	32	64	0.06	0.125
Δ*adeB*	64	128	16	16	0.06	0.125
Δ*adeJ*	64	128	32	64	0.06	0.125
